# Reliability issues in human brain temperature measurement

**DOI:** 10.1186/cc7943

**Published:** 2009-07-02

**Authors:** Charmaine Childs, Graham Machin

**Affiliations:** 1 Brain Injury Research Group, School of Translational Medicine, University of Manchester, Clinical Sciences Building, Salford Royal NHS Foundation Trust, Stott Lane, Salford, M6 8HD, UK; 2 Head of Temperature Standards, Temperature Group, National Physical Laboratory, Teddington, Middlesex, TW11 0LW, UK

## Abstract

**Introduction:**

The influence of brain temperature on clinical outcome after severe brain trauma is currently poorly understood. When brain temperature is measured directly, different values between the inside and outside of the head can occur. It is not yet clear if these differences are 'real' or due to measurement error.

**Methods:**

The aim of this study was to assess the performance and measurement uncertainty of body and brain temperature sensors currently in use in neurocritical care. Two organic fixed-point, ultra stable temperature sources were used as the temperature references. Two different types of brain sensor (brain type 1 and brain type 2) and one body type sensor were tested under rigorous laboratory conditions and at the bedside. Measurement uncertainty was calculated using internationally recognised methods.

**Results:**

Average differences between the 26°C reference temperature source and the clinical temperature sensors were +0.11°C (brain type 1), +0.24°C (brain type 2) and -0.15°C (body type), respectively. For the 36°C temperature reference source, average differences between the reference source and clinical thermometers were -0.02°C, +0.09°C and -0.03°C for brain type 1, brain type 2 and body type sensor, respectively. Repeat calibrations the following day confirmed that these results were within the calculated uncertainties. The results of the immersion tests revealed that the reading of the body type sensor was sensitive to position, with differences in temperature of -0.5°C to -1.4°C observed on withdrawing the thermometer from the base of the isothermal environment by 4 cm and 8 cm, respectively. Taking into account all the factors tested during the calibration experiments, the measurement uncertainty of the clinical sensors against the (nominal) 26°C and 36°C temperature reference sources for the brain type 1, brain type 2 and body type sensors were ± 0.18°C, ± 0.10°C and ± 0.12°C respectively.

**Conclusions:**

The results show that brain temperature sensors are fundamentally accurate and the measurements are precise to within 0.1 to 0.2°C. Subtle dissociation between brain and body temperature in excess of 0.1 to 0.2°C is likely to be real. Body temperature sensors need to be secured in position to ensure that measurements are reliable.

## Introduction

In rodent models of cerebral ischaemia, small (1° to 2°C) increases in brain temperature significantly increase infarct volume [1-3]. In patients with brain damage, the same risks are thought to apply; temperatures within the febrile range are widely perceived to increase the risk of a worse patient outcome [4-6]. However, as there remains no clear evidence for a causal relation, the influence of brain temperature on acute clinical physiology, particularly on intracranial pressure (ICP), is currently poorly understood [7].

The significance of raised brain temperature on outcome in neurocritical care (NCC) patients remains controversial [8,9]. Studies can be cited that point in either direction: that a high temperature [10] or a low temperature [11] is an indicator of poor prognosis. As brain temperature is not measured routinely in NCC, body temperature is used as a surrogate for intracranial temperature. However, differences between brain and body temperature can occur which are often very subtle (often smaller than 0.5°C) [12,13], so the performance or positioning of the temperature sensor placed within the brain or peripheral sites (e.g., rectum) becomes important. In short, are the subtle differences observed between brain and body temperature 'real' or due to measurement error? The aim of this study was to assess performance and measurement uncertainty of the body and brain temperature sensors currently used in NCC.

## Materials and methods

### Evaluation of temperature sensors

The performance of three commonly used temperature sensors for brain and body temperature measurement was undertaken as a service evaluation. Ethical approval and patient consent were therefore not sought. The sensors were: brain type 1 (Raumedic, Neurovent-P-T, Eden Medical, Midlothian, Scotland); brain type 2 (Camino 110-4BT, Integra Neurosciences, Andover, UK); and a widely used (body type) general purpose probe (Thermistor 400 series, Mallinckrodt Medical, Tyco Healthcare, Gosport, UK). Brain and body type sensors were of a thermistor type [14] but of different dimensions. Sensors were selected randomly from different batches, obtained from the manufacturers at different time intervals.

### Sensor calibration

The temperature measurement evaluation of these probes was undertaken through the use of ultra-stable organic triple-point temperature references of 26.862°C (based on diphenyl ether (DPE) and 36.314°C (based on ethylene carbonate (EC)) [15]. These temperature references were calibrated directly traceable to the International Temperature Scale of 1990 [16] as realised at the National Physical Laboratory [17] with an expanded uncertainty of ± 0.005°C [18]. A detailed test protocol was established so that the sensors could be evaluated on a common objective basis. Six of each of the three temperature sensor types under test were calibrated separately (n = 18) in each triple-point cell (DPE and EC).

### Measurement uncertainty

Uncertainty values were calculated according to guidelines given in the internationally accepted *Guide to the Expression of Uncertainty in Measurement *[18]. Briefly, the uncertainty evaluation involves identifying and quantifying all the individual sources of uncertainty of the temperature measurement. Two classes of uncertainty are generally identified. Those obtained by statistical means are known as type A (e.g., the standard deviation of the mean) and those obtained by methods other than statistical analysis are known as type B, often known as systematic effects (e.g., the temperature uncertainty of the fixed-point cell itself, the short-term repeatability of the individual sensors and the sensor batch repeatability; ie, quantifying the agreement of the six sensors of each type).

In addition, the effect of a change in sensor position on the measurement (immersion test) was assessed. In this test the three types of sensors were immersed in turn into a column of liquid at a uniform temperature (at the base of the re-entrant well of the triple-point cell) and then withdrawn in 1 cm increments up the column. The immersion test was undertaken over a period of 15 minutes for one sensor of each sensor type. A further source of uncertainty was obtained by assessing the change in temperature values using a different readout system, that is, performing the measurement uncertainties but using a different bedside data acquisition system (of the type used on the NCC unit). These individual uncertainties are then processed according to internationally agreed methodology to determine the standard uncertainties (ie, one standard uncertainty). For a fuller explanation of uncertainties and how to develop rigorous uncertainty 'budgets' the interested reader is referred to reference [18].

## Results

Average differences between the temperature of the DPE triple-point cell (the reference source, nominally 26.8°C) and the temperature sensors were +0.11°C (brain type 1), +0.24°C (brain type 2) and -0.15°C (body type), respectively. For the EC triple-point cell (temperature reference source nominally 36.3°C) the differences were -0.02°C (brain type 1), +0.09°C (brain type 2) and -0.03°C (body type). Repeat calibrations the following day confirmed that these results were within the calculated uncertainties.

**Figure 1:**
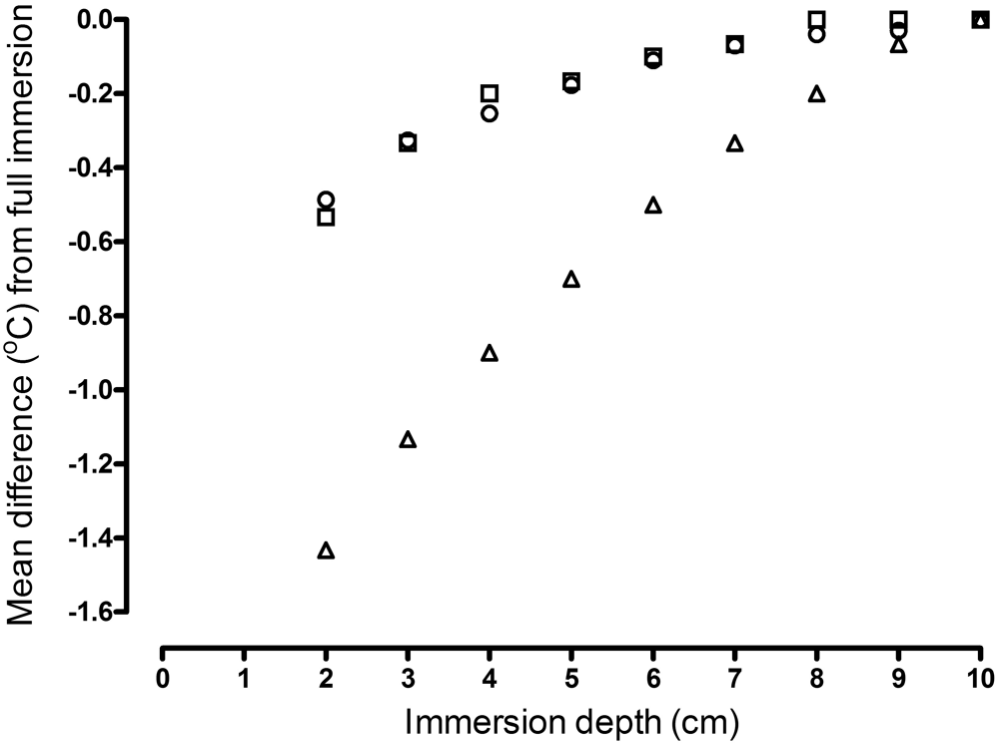
Effects of immersion on temperature sensor readings. Mean difference between temperature sensors (circles = brain type 1; squares = brain type 2; triangles = body type) and ethylene carbonate triple-point temperature (26°C) reference source with sensor at intervals of 1 cm from base (10 cm depth of immersion) of re-entrant well. As an example for the body type sensor, full immersion is at 10 cm. Withdrawing the sensor 4 cm up the re-entrant well changes the immersion depth to 6 cm and a corresponding change in the temperature reading of 0.5°C.

The results of the immersion tests are given in Figure 1. The temperature performance of the body type sensor is clearly influenced by the sensor position. A change in sensor position (movement of the sensor up from the base (depth 10 cm) of the re-entrant well) of between 4 to 8 cm results in a measured difference between the sensor and reference temperature of -0.5 to -1.4°C. The expanded uncertainty for the calibration, at both triple-point temperatures, of sensors brain type 1, brain type 2 and body type were ± 0.18°C, ± 0.10°C and ± 0.12°C, respectively.

## Discussion

In previous work undertaken at this Centre (Intensive Care Unit of Salford Royal NHS Foundation Trust, Salford UK) small temperature differences observed between body and brain sites [19] led us to question whether such differences were due to real physiological events or could in part/wholly be due to sensor performance. This study specifically investigates the sensor aspects of this issue.

In this study of the reliability of body and brain temperature sensors used in the management of patients with severe brain trauma it was necessary to undertake rigorous quantification of all possible sources of measurement uncertainty. We considered the magnitude of each source of uncertainty separately to produce an overall assessment according to agreed international methods [18].

For the limited number of sensors tested, the performance was within the manufacturer's specification for temperatures at near body temperature; all (brain and body) sensors were within 0.1°C of the 36°C reference temperature source. The body-type sensors tested at below normal body temperature (reference source 26.8°C) did not perform as well as the brain sensors (measurement error +0.24°C).

One difference between the brain and body type sensors was that a change in position (immersion depth) of the body type sensor caused a change in the temperature readout (Figure 1). Such an immersion effect using the body temperature sensor needs to be considered for its potential clinical implications, that is, by changing the sensor position by 4 cm a clinically relevant temperature difference occurred. This suggests that the reliability of a patient's temperature could be in doubt if the sensor is dislodged from its original *in situ *position. This effect was not observed with the brain temperature sensors, suggesting that the brain temperature sensors tested in this study are less susceptible to ambient temperature than were the body type sensors.

The results of this study therefore provide important information of relevance to clinicians interested in the potential significance of changes in the relation between brain and body temperature [19].

## Conclusions

The result of this service evaluation of sensors in current use for the measurement of brain and body temperature in critically ill patients reveals that the temperature sensors (brain and body) are fundamentally accurate and the measurements are precise to within 0.1 to 0.2°C. Subtle dissociation between brain and body temperature in excess of 0.1 to 0.2°C is likely to be real. This should be confirmed with a larger study. Good clinical practice in human thermometry is to ensure that body temperature sensors are checked for secured positioning.

## Key messages

• The brain temperature sensors are fundamentally accurate and the measurements are precise to within 0.1 to 0.2°C.

• When seeking to determine the clinical significance of these subtle temperature differences it is essential that the sensors are firmly secured in position.

• Observed dissociation between brain and body temperature in excess of 0.1 to 0.2°C are likely to be 'real' and not due to sensor performance.

## Abbreviations

DPE: diphenyl ether; EC: ethylene carbonate; ICP: intracranial pressure; NCC: neurocritical care.

## Competing interests

The authors declare that they have no competing interests.

## Authors' contributions

CC conceived the idea for the study, participated in the sensor calibrations and evaluations and drafted the manuscript. GM provided the organic fixed-point reference sources, participated in the sensor calibrations, calculated the sensor measurement uncertainties and helped to draft the manuscript. The authors are equally responsible for the final manuscript.
